# Management of Localized Prostate Cancer by Focal Transurethral Resection of Prostate Cancer: An Application of Radical TUR-PCa to Focal Therapy

**DOI:** 10.1155/2012/564372

**Published:** 2012-05-22

**Authors:** Masaru Morita, Takeshi Matsuura

**Affiliations:** ^1^Department of Urology, Kounaizaka Clinic, 1917-3 Asakura-Hei, Kochi 780-8063, Japan; ^2^Department of Urology, Matsubara Tokushukai Hospital, 7-13-26 Amami-Higashi, Matsubara, Osaka 580-0032, Japan

## Abstract

*Background*. We analyzed radical TUR-PCa against localized prostate cancer. *Patients and Methods*. Seventy-nine out of 209 patients with prostate cancer in one lobe were studied. Patients' age ranged from 58 to 91 years and preoperative PSA, 0.70 to 17.30 ng/mL. In other 16 additional patients we performed focal TUR-PCa. Patients' age ranged from 51 to 87 years and preoperative PSA, 1.51 to 25.74 ng/mL. *Results*. PSA failure in radical TUR-PCa was 5.1% during the mean follow-up period of 58.9 months. The actuarial biochemical non-recurrence rate was 98.2% for pT2a and 90.5% for pT2b. Bladder neck contracture occurred in 28 patients (35.4%). In 209 patients, pathological study revealed prostate cancer of the peripheral zone near the neurovascular bundle bilaterally in 25%, unilaterally in 39% and no cancer bilaterally in 35%, suggesting the possibility of focal TUR-PCa. Postoperative PSA of 16 patients treated by focal TUR-PCa was stable between 0.007 and 0.406 ng/mL at 24.2 months' follow-up. No patients suffered from urinary incontinence. Bladder neck contracture developed in only 1 patient and all 5 patients underwent nerve-preserving TUR-PCa did not show erectile dysfunction. *Conclusion*. Focal TUR-PCa was considered to be a promising option among focal therapies against localized prostate cancer.

## 1. Introduction

Current standard radical surgery [1–3] against localized prostate cancer (PCa) has possible risks to disturb urinary continence or erectile function because they target the whole prostate. Many operative procedures [4–6] were introduced to improve the recovery of postoperative sexual function and urinary incontinence, such as bladder neck suspension or reconstruction, reconstruction of the rhabdosphincter, periurethral suspension of the dorsal vein complex/urethral complex and preservation of the neurovascular bundle to preserve erectile function [7]. But all these have failed to solve the problems completely until now. Irradiation therapy such as brachytherapy [8], three-dimensional conformal radiation therapy *（*3D-CRT*）* [9], or intensity-modulated radiation therapy (IMRT) [10] cannot completely prevent urinary incontinence, intestinal damage, or erectile dysfunction as well.

 As the number of patients with low-volume, low-grade localized prostate cancer increased after the introduction of PSA into health check-up program, less invasive focal therapy has been proposed because of possible advantages of both cancer control and quality of life. Cryotherapy [11, 12] and high-intensity focused ultrasound (HIFU) [13, 14] are current main procedures of focal therapy but are still considered experimental.

 We previously reported that radical transurethral resection of prostate cancer (RTUR-PCa) could be a radical therapy against localized prostate cancer [15]. We then referred to the possibility of focal transurethral resection of prostate cancer (FTUR-PCa), the procedure in which we mainly resected the affected lobe of the prostate [16]. We made a retrospective analysis of RTUR-PCa against localized prostate cancer to evaluate whether FTUR-PCa could be a valid focal therapy. We here report our result of FTUR-PCa, too.

## 2. Patients and Methods

### 2.1. Cases of RTUR-PCa as a Database to Estimate FTUR-PCa

Between December 2003 and July 2009, a total of 261 RTUR-PCa were performed under spinal anesthesia in 209 patients with localized prostate cancer. Clinical stages were determined according to the UICC TNM staging system of 1997. We performed bone scintigraphy and computerized tomography for the purpose of metastatic workup in patients who had initial PSA levels of 20 ng/mL or more. And we recommended the patients who had PSA level between 10 and 20 ng/mL and higher Gleason score to undertake such examinations with some of them having refused to take. We informed the patients that the procedure was not a standard radical surgery, and those who refused this procedure were excluded from the study. We also excluded patients who might not tolerate standard transurethral resection of the prostate (TURP). Patients who gave the written informed consent were eligible for the study in the order they were given a diagnosis of localized prostate cancer. Institutional review board approved the TUR-PCa program after a preliminary study.

### 2.2. Retrospective Analysis to Evaluate the Efficacy and Safety of FTUR-PCa

 We thought that the most appropriate indication of focal therapy should be localized prostate cancer in one lobe. We reviewed RTUR-PCa cases to find patients to match such criteria. Seventy-nine of the above 209 RTUR-PCa patients diagnosed to have prostate cancer in one side of the lobes were included in the present study. In 74 out of these 79 patients, cancer was detected by ultrasound-guided transrectal needle biopsy, and in the other 5 patients prostate cancer was incidentally detected after TURP for benign prostate hyperplasia (BPH). We obtained a total of 14 biopsy samples per case from the peripheral and transition zone including far lateral part, dividing the prostate into base (2 cores), upper middle part (2 cores), lower middle part (6 cores), and apex (4 cores), and we marked at the dorsal end to obtain tumor maps. In the patients underwent TURP; after resecting most of the transition and central zone, we made a slightly deeper resection dividing the residual thin transition zone and peripheral zone into 6 parts and collected the resected specimens separately to identify the affected sites by pathological examination (advanced TURP) [15].

 We performed 93 RTUR-PCa in 79 patients under spinal anesthesia. Patients ranged from 58 to 91 years old (mean ± SD, 73.9 ± 6.6; median, 74.0) and preoperative PSA, 0.70 to 17.30 ng/mL (mean ± SD, 5.77 ± 3.51; median, 4.57). Chlormadinone acetate was administered for a mean period of 4.6 months in 52 patients.

### 2.3. Focal TUR-PCa

 Between July 2007 and September 2011, we performed FTUR-PCa in 16 patients. FTUR-PCa includes two different procedures: in one procedure we radically resect the affected one lobe with unaffected lobe being resected as advanced TURP for BPH (one lobe radical TUR), and in the other procedure we radically resect both lobes except for the prostate tissues near the neurovascular bundle (nerve sparing radical TUR) based on the tumor mapping. Patients ranged from 51 to 87 years old (mean ± SD, 68.9 ± 9.6; median, 70.0) and preoperative PSA, 1.51 to 25.74 ng/mL (mean ± SD, 7.87 ± 6.35; median, 6.19). Chlormadinone acetate was not administered in this group.

### 2.4. Surgical Procedure and Followup

 One urologic surgeon (M. Morita) performed all operations. We used a standard TURP setup with an irrigation pressure of 80 cm H_2_O and an irrigation rate of 250 mL/min using D-sorbitol solution. After resecting almost all the transition and central zone, we tried to resect and fulgurate the peripheral zone completely especially where cancer was detected by biopsy. The resection was performed as deep as adipose tissue was identified and as distal as the external sphincter was identified. But we did not resect prostate tissues until adipose tissue was exposed all around the operative field. We aggressively fulgurated the area adjacent to where adipose tissue was exposed because the remaining prostate tissue could be considered a thin layer. We especially paid attention not to distend the bladder too much to prevent a high irrigation pressure and resultant TUR syndrome. Special attention was also paid to avoid injury to Santorini's plexus and the rectum. The procedure was started from the 12 o'clock position, dividing the prostate into 6 parts, and resected specimens were separately collected from each part to examine the distribution of cancer. The seminal vesicle was partially resected at its attached part to the prostate between the 4 and 8 o'clock positions to determine the invasion of cancer. Finally the verumontanum was resected to achieve the complete resection of prostate tissue. A bag catheter was removed on the third postoperative day.

 Postoperative PSA was measured every two months starting two months after the operation. PSA failure was suspected when PSA levels showed a consecutive rise over 0.2 ng/mL. But when the PSA level reached a plateau between 0.2 and 1.0 ng/mL, we did not immediately think that the patients were in a treatment failure. This was also applied to the indication of the second RTUR-PCa. We evaluated stress urinary incontinence by asking patients the postoperative status of urinary leak on a cough or a sneeze and needs for urinary pads.

## 3. Results

### 3.1. Retrospective Analysis Based on the Results of Radical TUR-PCa

The mean follow-up period of 79 patients was 58.9 ± 17.0 months (mean ± SD; median, 60.5; range, 15–88). The operation time ranged between 65 and 120 minutes (mean ± SD, 79.9 ± 15.2; median, 80.0), and the resected tissue weight was between 5.0 and 37.0 grams (mean ± SD, 13.3 ± 6.4; median, 12.0). The preoperative PSA value was 5.77 ± 3.51 ng/mL (mean ± SD; median, 4.57; range, 0.70–17.3). Clinical stages were as follows: T1b, 28 cases; T1c, 47; T2, 4; and pathological stages: pT2a, 56 cases; pT2b, 21; pT3, 2 (Table 1). Gleason scores were: 4, 3 cases; 5, 3; 6, 27; 7, 28; 8, 12; 9, 6 (Table 2).

One patient died of cerebrovascular accident 15 months postoperatively with a low PSA value of 0.012 ng/mL. No patients died of prostate cancer. Sixty-four patients had stable PSA after the first operation. The second operation was required in 14 patients after a mean period of 16.4 months (mean ± SD, 16.4 ± 7.5; median, 16.0; range, 6.4 to 30.5) due to rising PSA levels. Resected tissue weight was between 5.0 and 12.0 grams (mean ± SD, 6.9 ± 1.6; median, 12.0). No cancer was detected by pathological examination in 3 patients. The second operation was required in 10 (40.0%) out of 25 patients before April 2006 but in only 4 (7.0%) out of 54 patients after that time, suggesting that there seemed to be a learning curve for the operative technique.

**Figure 1:**
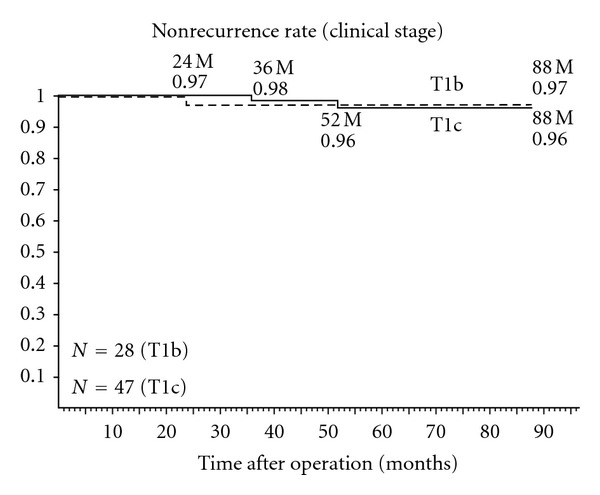
Actuarial biochemical non-recurrence rate of each clinical stage.

**Figure 2:**
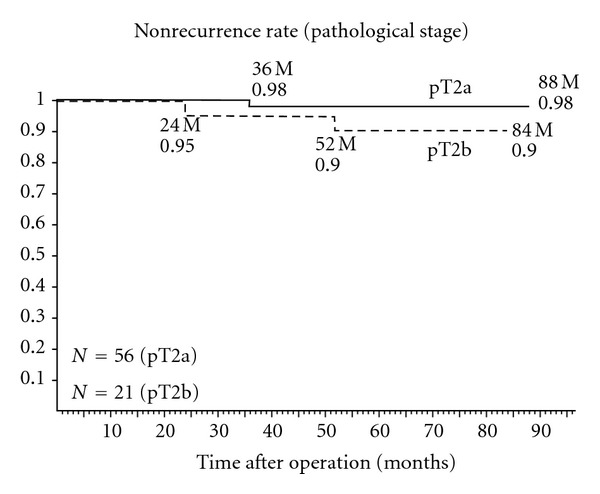
Actuarial biological non-recurrence rate of each pathological stage.

**Figure 3:**
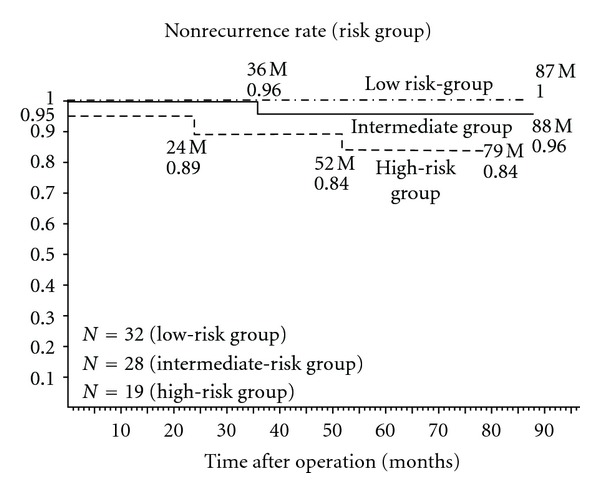
Actuarial biological non-recurrence rate of each risk group.

 At the final followup, there were 75 (94.9%) patients with stable PSA levels: PSA ≦ 0.01, 40 cases; ≦0.02, 12; ≦0.03, 5; ≦0.04, 5; ≦0.1, 5; ≦1.0, 8. PSA failure developed in 4 (5.1%) of the studied patients. Clinical stages were as follows: T1b, 1 case; T1c, 2; T2, 1; pathological stages: pT2a, 1 case; pT2b, 2; pT3, 1. In all cases studied, the actuarial biological non-recurrence rate was 96.4% for the clinical stage T1b and 95.7% for T1c at 58.9 months (Figure 1). PSA failure developed in one of 4 patients with stage T2 cancer. The actuarial biological non-recurrence rate was 98.2% for the pathological stage pT2a at 88 months and 90.5% for pT2b at 84 months (Figure 2). One of 2 patients with stage pT3 cancer developed PSA failure. Nonrecurrence rate of each risk group according to The D'Amico classification [17] is shown in Figure 3. PSA failure did not develop in the low-risk group (stage T1c, T2a, and PSA level ≦ 10 ng/mL and Gleason score ≦ 6) of 32 patients. Biological non-recurrence rate was 96.4% in the intermediate-risk group (stage T2b or Gleason score of 7 or 10 < PSA level ≦ 20 ng/mL) of 28 patients and 84.2% in the high-risk group (stage T2c or PSA level > 20 ng/mL or Gleason score ≧ 8) of 19 patients, respectively.

To evaluate the distribution of prostate cancer near the neurovascular bundle, we studied the result of pathological examination of resected samples in 209 RTUR-PCa patients. Cancer was detected at the 4 to 6 o'clock position in 44 patients (21%), at the 6 to 8 o'clock position in 38 patients (18%), bilaterally (at the 4 to 8 o'clock position) in 74 patients (35%) and was not detected bilaterally in 53 patients (25%).

### 3.2. Result of FTUR-PCa

 Concerning the 16 patients underwent FTUR-PCa, the follow-up period ranged from 3 to 53 months (mean ± SD, 24.2 ± 15.4; median, 22.0), operation time 80 to 120 minutes (mean ± SD, 92.5 ± 11.6; median, 90.0), and resected tissue weight 12 to 30 grams (mean ± SD, 21.0 ± 4.9; median, 20.0). Postoperative PSA was stabilized between 0.07 and 0.406 ng/mL (mean ± SD, 0.119 ± 0.111; median, 0.090). The Gleason scores were as follows: 5, 1 case; 6, 4; 7, 8; 8, 1; 9, 2. Clinical stages were T1c in all patients, and pathological stages were as follows: pT2a, 10 cases and pT2b, 6. There were 11 cases underwent one lobe radical TUR and 5 cases underwent nerve sparing radical TUR. Eight (72.7%) out of 11 patients underwent one lobe radical TUR had the postoperative pathological stage of pT2a.

### 3.3. Operative Complications

 TUR syndrome (systolic hypotension or electrolyte abnormality which needs repeated correction to keep vital signs stable) and perioperative bleeding (bleeding during the procedure or bladder tamponade that requires blood transfusion) did not develope.

As for the postoperative complication of RTUR-PCa, urinary incontinence was seen in about half of the patients when a bag catheter was removed on the third postoperative day. Incontinence soon improved until the third postoperative week, and no patients complained of stress urinary incontinence at all after the third postoperative month. Bladder neck contracture developed in 28 patients (35.4%; Grade IIIa by Clavien's classification) mostly three to four months postoperatively. Other complications included one pubic osteitis (1.3%; Grade II) and one acute epididymitis (1.3%; Grade II). Erectile function was preserved after the operation in 9 (50.0%) of the evaluated 18 sexually active patients.

 In FTUR-PCa, bladder neck contracture developed in only 1 out of 16 patients. We experienced no stress urinary incontinence, and erectile function was preserved in the all 5 patients underwent nerve-sparing TUR.

## 4. Discussion

### 4.1. Limitations of the Accuracy of Preoperative Staging

 Out of 79 patients with the clinical stage of T2a, 56 (70.9%) patients were finally diagnosed to have the pathological stage of pT2a disease with PSA non-recurrence rate of 98.2% at a mean follow-up period of 58.9 months. These results may support that FTUR-PCa is a promising procedure. But it may be desirable to make the cancer distribution in the prostate more accurate to select proper candidates of the procedure. Only 27.3% to 35.1% of patients with unilateral cancer on biopsy are reported to have cancer in one lobe on radical prostatectomy samples [18, 19]. Crawford et al. report that the concordant rate of laterality could be 76% on saturation biopsy using a template [20]. And accuracy of biopsy is reported better in transperineal approach with a template than transrectal biopsies [21]. From the result of pathological studies in our 209 RTUR-PCa, bilateral preservation of the neurovascular bundle was thought to be possible in as many as 25% of the patients and unilateral preservation in 39%. Current biopsy procedures may be insufficient to predict cancer foci near the neurovascular bundle. Neurovascular bundle reserving TUR, unilateral or bilateral, is worth trying based on biopsy information, because TUR-PCa is possible to repeat if postoperative PSA tends to rise gradually.

### 4.2. Comparison with Other Focal Therapies and Rationales of FTUR-PCa

 The number of patients with low-volume, low-grade localized prostate cancer has increased after the introduction of PSA into health check-up program [18, 22]. Ablation of only main cancer lesion (index cancer) in one lobe by HIFU or cryotherapy is reported not to affect the prognosis as much [23, 24] because the second lesion, if it exists, is usually as small as less than 0.5 mL [25, 26]. But these procedures have some serious drawbacks concerning the selection of patients. Current methods of biopsy cannot always predict another lesion to be treated, and followup PSA criteria have not been established yet. Furthermore, pathological samples cannot be obtained, resulting in the inadequate final pathological diagnosis. On the other hand, FTUR-PCa is possible to search the second cancer focus after the radical resection of main index cancer in one lobe and advanced TUR in another lobe [27], resulting in the accurate diagnosis of Gleason scores and the pathological stage. And the second TUR may be possible, when necessary, using PSA as an indicator of postoperative follow-up and cancer recurrence. We therefore consider that FTUR-PCa could be another possible procedure of focal therapy against prostate cancer overcoming the drawbacks of HIFU and cryotherapy. Our present result of FTUR-PCa seems satisfactory in cancer control, urinary continence, and erectile function, though the follow-up period is as short as 24.2 months in a very small number of patients.

 It is still controversial to support or not a mass screening of prostate cancer because screening may lead to overdiagnosis and overtreatment. Active surveillance policy or watchful waiting, which is an ultimate noninvasive procedure, is then accepted to care for the patients with low-risk cancer [28, 29]. But active surveillance seems still difficult to select a suitable patient, and the patient may feel anxiety about cancer progression. Focal therapy is less invasive compared to current standard treatment procedures such as radical prostatectomy or irradiation therapy and may be a treatment option with satisfactory cancer control and quality of life. FTUR-PCa can expand the possibility of focal therapy, and we think it is one of the feasible procedures to solve the problems of overdiagnosis and overtreatment caused by PSA screening of prostate cancer.

### 4.3. Possible Risk of FTUR-PCa and Some Other Considerations

 Dissemination of cancer cells may occur during TUR, but the effect on the clinical outcome is controversial [30–32]. Our previous report could not find any adverse effects on the prognosis of the studied patients [15, 16]. RTUR-PCa can eradicate cancer cells like other current standard radical therapy because repeated TUR is possible, and improvement of the surgical results will be expected.

 Extravasation of irrigation fluid is sure to occur during the operation, but there were no patients in whom water intoxication developed with the lowest irrigation pressure, and postoperative serum electrolytes were kept normal. At present much safer operation is possible with the use of a bipolar TUR system. The most frequent postoperative complication was bladder neck contracture occurred in 35.4% of patients 3 to 4 months after surgery. This had been anticipated because of aggressive bladder neck resection to achieve radicality. It was easily treated by optical urethrotomy under caudal block on a day-surgery basis. In FTUR-PCa, occurrence of bladder neck contracture is expected to be low because it is a radical procedure in only one affected lobe with advanced TUR in the other lobe.

 The effect of chlormadinone acetate on postoperative PSA must be considered in the present study. We could not find any reports that describe the duration of the suppressive effect of chlormadinone acetate in patients with prostate cancer. But in patients with prostate hyperplasia 50 mg/day of chlormadinone acetate given for 16 weeks, PSA levels are reported to return to the baseline levels 32 weeks after discontinuation [33]. In the present study the effect of preoperative hormonal therapy on the most recent PSA levels, therefore, can be minimal or negligible.

## 5. Conclusion

We could get a satisfactory cancer control with less invasive procedure of RTUR-PCa and FTUR-PCa. Although the results of long-term followup with more cases need to be studied, the procedure we report here could be a potential option of focal therapy against localized prostate cancer with minimum adverse effect on urinary continence and erectile function.

## Figures and Tables

**Table 1:** Results of RTUR-PCa grouped by pathological stage.

	*Total patients*		*Patients treated with 1 operation*				*Patients treated with 2 operations*			
Pathological stage	No. of patients	Preop PSA Mean (SD) Median (range)	No. of patients	Patients with stable PSA after TUR		No. of PSA failures	No. of patients	Patients with stable PSA after TUR		No. of PSA failures
				No. of patients	Latest PSA Mean (SD) Median (range)			No. Patient	Latest PSA Mean (SD) Median (range)	
pT2a	56	5.68 (3.42) 4.55 (0.70–17.30)	46	46	0.087 (0.191) 0.010 (0.001–0.897)	0	10	9	0.040 (0.078) 0.013 (0.003–0.258)	1
pT2b	21	5.44 (3.10) 4.33 (1.55–14.90)	17	17	0.019 (0.019) 0.010 (0.001–0.074)	0	4	2	0.001–0.008 (Range)	2
pT3	2	7.41–16.36 (Range)	2	1	0.001	1	0	—	—	0

**Table 2:** Results of RTUR-PCa grouped by Gleason's score.

	*Total patients*		*Patients treated with 1 operation*				*Patients treated with 2 operations*			
Gleason score	No. of patients	Preop PSA Mean (SD) Median (range)	No. of patients	Patients with stable PSA after TUR		No. of PSA failurse	No. of patients	Patients with stable PSA after TUR		No. of PSA failures
				No. of patients	Latest PSA Mean (SD) Median (range)			No. of patients	Latest PSA Mean (SD) Median (Range)	
4	3	4.59 (0.85) 4.41 (3.65–5.70)	3	3	0.064 (0.074) 0.016 (0.008–0.168)	0	0			
5	3	5.01 (2.85) 3.96 (2.17–8.90)	3	3	0.009 (0.006) 0.009 (0.002–0.016)	0	0			
6	27	4.52 (2.38) 4.33 (1.55–11.54)	24	24	0.096 (0.184) 0.017 (0.001–0.685)	0	3	3	0.010 (0.008) 0.008 (0.001–0.021)	0
7	28	6.64 (3.76) 4.80 (2.01–16.36)	21	21	0.065 (0.192) 0.006 (0.001–0.897)	0	7	6	0.009 (0.005) 0.010 (0.001–0.016)	1
8	12	5.54 (3.53) 4.52 (0.70–12.40)	10	10	0.038 (0.102) 0.002 (0.001–0.344)	0	2	1	0.025	1
9	6	8.84 (4.55) 7.77 (3.50–17.30)	4	3	0.027 (0.011) 0.033 (0.011–0.036)	1	2	1	0.258	0
